# Retrospective agreement and consent to neurocritical care is influenced by functional outcome

**DOI:** 10.1186/cc9210

**Published:** 2010-07-30

**Authors:** Ines C Kiphuth, Martin Köhrmann, Joji B Kuramatsu, Christoph Mauer, Lorenz Breuer, Peter D Schellinger, Stefan Schwab, Hagen B Huttner

**Affiliations:** 1 Department of Neurology, University of Erlangen, Schwabachanlage 6, 91054 Erlangen, Germany

## Abstract

**Introduction:**

Only limited data are available on consent and satisfaction of patients receiving specialized neurocritical care. In this study we (i) analyzed the extent of retrospective consent to neurocritical care--given by patients or their relatives--depending on functional outcome one year after hospital stay, and (ii) identified predisposing factors for retrospective agreement to neurocritical care.

**Methods:**

We investigated 704 consecutive patients admitted to a nonsurgical neurocritical care unit over a period of 2 years (2006 through 2007). Demographic and clinical parameters were analyzed, and the patients were grouped according to their diagnosis. Functional outcome, retrospective consent to neurocritical care, and satisfaction with hospital stay was obtained by mailed standardized questionnaires. Logistic regression analyses were calculated to determine independent predictors for consent.

**Results:**

High consent and satisfaction after neurointensive care (91% and 90%, respectively) was observed by those patients who reached an independent life one year after neurointensive care unit (ICU) stay. However, only 19% of surviving patients who were functionally dependent retrospectively agreed to neurocritical care. Unfavorable functional outcome and the diagnosis of stroke were independent predictors for missing retrospective consent.

**Conclusions:**

Retrospective agreement to neurocritical care is influenced by functional outcome. Especially in severely affected stroke patients who cannot communicate their preferences regarding life-sustaining therapy, neurocritical care physicians should balance the expected burdens and benefits of treatment to meet the patients' putative wishes. Efforts should be undertaken to identify predictors for severe disability after neurocritical care.

## Introduction

In the past, physicians did not routinely seek permission from patients before initiating diagnostic and therapeutic procedures, regardless of the risk [1]. However, in recent years, emphasis has been shifted from physician sovereignty to patient autonomy, obliging physicians to expect and encourage patient participation in decision making after having given them all available relevant information, thus obtaining the patient's informed consent to perform the given procedure [2,3]. Neurologic patients in need of intensive care, however, may not be capable of participating in the informed-consent process, because of reduced consciousness or severe aphasia. Moreover, further clinical deterioration and complications may occur within the first hours after admission. Rapid identification and implementation of a suitable legal representative for participation in the consent process can be difficult and, in some instances, not feasible. Hence, in life-threatening situations, physicians perform procedures without consent, assuming that most individuals would assent to be treated in this situation. This approach has been widely discussed and agreed on [1-3]. However, taking into account the possibility of an unfavorable outcome after neurointensive care, would patients really agree to a treatment that increases the chance for survival with, conversely, the potential perspective of severe disability with the need for constant nursing care or even delayed death?

Our objective was to assess patients' retrospective willingness to undergo neurocritical care, given that functional outcome may be very poor. Furthermore, patients and relatives, regardless of the functional outcome, were asked whether they were satisfied with their treatment on our neurocritical care unit.

## Materials and methods

### Patients and Setting

Between January 2006 and December 2007, 796 neurologic patients were admitted to our 10-bed neurocritical care unit (tertiary University Hospital). As we aimed to investigate the retrospective consent to specialized neurocritical care, we excluded 92 patients from this analysis because of (a) being temporarily monitored only after neuroradiologic procedures (*n *= 21), (b) representing outsourced patients from general ICUs (*n *= 42), or (c) being lost to follow up (*n *= 29). A total of 704 patients remained eligible for the final analysis. The institutional review board approved the study, and consent was obtained in written or oral form from all patients or their relatives/legal guardians.

### Data collection

We obtained age, diagnoses, and medical history by reviewing the patients' hospital charts and institutional electronic databases. Patients were grouped according to their neurologic diagnoses (1, ischemic stroke; 2, intracerebral hemorrhage (ICH); 3, subarachnoid hemorrhage (SAH); 4, meningoencephalitis; 5, epileptic seizures; 6, Guillain-Barré syndrome (GBS) and myasthenia gravis (MG); 7, neurodegenerative diseases and encephalopathy; 8, cerebral neoplasm; and 9, intoxication).

Patients or their relatives were contacted by using a mailed standardized questionnaire, which was answered by either the patient or the next of kin or the legal guardian (the legal guardianship was reassessed in regular intervals by the responsible courts). In all cases in which this questionnaire did not return within 6 weeks, a structured phone interview was conducted with the patients or their closest relatives. The telephone interviews were performed by a stroke physician who was trained and certified for data collection on disability, quality of life, and the modified Rankin Scale (mRS). Consent to participate in this study was obtained in written or oral form from all patients or their relatives/legal guardians. Because of functional impairment or death, a proportion of patients were not able to answer themselves (see Table 1).

**Table 1:** Retrospective consent

mRS	Patient replied (*n*/%)	Relatives replied (*n*/%)	*P *value
0	57 (100%)	0	<0.0001
1	98 (100%)	0	<0.0001
2	36 (100%)	0	<0.0001
3	44 (73.3%)	16 (26.7%)	<0.0001
4	50 (79.4%)	13 (20.6%)	<0.0001
5	9 (9.2%)	89 (90.8%)	<0.0001
6	0	292 (100%)	<0.0001
Total	294 (41.8%)	410 (58.2%)	

Numbers of patients *versus *relatives who answered to the question of retrospective consent for neurocritical care. Data are separately given, depending on functional outcome of patients.

mRS, modified Rankin Scale; *n*, number.

### End-point definition

Patients were asked to answer four questions: (a) functional status (expressed as mRS) before hospital admission, and (b) functional outcome (expressed as mRS) 12 months after hospital stay. Furthermore, patients were asked (c) if they retrospectively agreed with the treatment (that is, whether they would again consent to neurocritical care, given the experienced functional outcome 1 year after hospital stay). Consent was defined as the retrospective approval of the applied life-saving emergency procedures (for example, intubation, placement of ventricular drains) on admission to the neurocritical care unit (thereby explaining that withdrawal of consent would probably have correlated with rapid clinical worsening and the probability of early death). The possibility of answering the question of retrospective consent to neurocritical care was dichotomized. Finally, (d) patients were asked whether they were satisfied with their hospital stay. Satisfaction was defined as general contentment with the neuro-ICU (NICU) stay, wilfully neglecting the functional outcome (that is, contentment with how the patient and family members were covered by the staff, educated with regard to the prognosis and future course of the disease, and experienced decision making). The questionnaire provided five possible answers: very satisfied, satisfied, neither satisfied nor dissatisfied, dissatisfied, and very dissatisfied. The answers 'very satisfied' and 'satisfied' were categorized as satisfied, whereas 'neither satisfied nor dissatisfied,' 'dissatisfied,' and 'very dissatisfied' were scored as dissatisfied.

Functional outcome was defined as favorable (mRS 0 to 1), mild to moderate disability (mRS 2 to 3), severe disability (mRS 4 to 5), and dead (mRS 6) [4], and also as independent (mRS 0 to 2) versus dependent or dead (mRS 3 to 6).

### Statistical analysis

Statistical analyses were performed by using the SPSS 17.0 software package (SPSS Inc., Chicago, IL). Statistical tests were two-sided, and the significance level was set at α = 0.01. The distribution of the data was assessed with the Kolmogorov-Smirnov test. Continuous and categoric variables are expressed as median and range, or as percentage, as appropriate. Proportions between two groups were compared by using the χ^2 ^test, Fisher's Exact test, or the Mann-Whitney *U *test, as appropriate.

One stepwise forward-inclusion multivariate logistic regression model was calculated to determine parameters that independently predisposed for retrospective consent. Those parameters that showed at least a trend in univariate testing (*P *< 0.1) were included into the multivariate analysis. The parameter mRS was entered as a nominal variable. Interaction terms did not reveal significant interaction between the variables.

## Results

The demographic and clinical characteristics of the analyzed 704 patients, as well as overall amount of consent and satisfaction, are given in Table 2. Of 241 patients with cerebral ischemia, 14 received decompressive surgery for malignant middle cerebral artery infarction, 11 of whom retrospectively consented to neurointensive care. Of 205 patients with ICH, hematoma evacuation was performed in 27 patients, nine of whom retrospectively consented.

**Table 2:** Demographic and clinical data

	All	Ischemia	ICH	SAH	Meningoencephalitis	Epilepsy	GBS/MG	Neurodeg./ Enceph.	Cerebral neoplasm	Intox.
*n *(%)	704	241 (34.2)	205 (29.1)	37 (5.3)	47 (67)	86 (12.2)	24 (3.4)	20 (2.8)	19 (2.7)	25 (3.6)

Age (median, range)	67 (18-95)	72 (21-93)	70 (35-95)	56 (19-84)	63 (27-85)	59 (18-93)	58 (23-78)	66 (23-85)	65 (39-78)	53 (29-78)
Female sex (*n*, %)	328 (46.6)	107 (44.4)	95 (46.3)	19 (51.4)	24 (51.1)	42 (48.8)	13 (54.2)	13 (65.0)	7 (36.8)	8 (32.0)
Mechanical ventilation (*n*, %)	447 (63.5)	143 (59.3)	141 (68.8)	20 (54.1)	40 (85.1)	44 (51.2)	15 (62.5)	17 (85.0)	7 (36.8)	20 (80.0)
Preadmission mRS 0-2 (*n*, %)	628 (89.2)	226 (93.7)	180 (87.8)	34 (91.9)	42 (89.4)	63 (73.3)	23 (95.8)	9 (45.0)	6 (31.6)	45 (96.0)
Consent of all patients (*n*, %)	361 (51.3)	98 (40.7)	76 (37.1)	23 (62.1)	38 (80.9)	62 (72.1)	19 (79.2)	11 (55.0)	14 (73.7)	20 (80.0)
Satisfaction (*n*, %)	643 (91.3)	224 (92.9)	195 (95.1)	35 (94.6)	45 (95.7)	65 (75.6)	22 (91.7)	18 (90.0)	16 (84.2)	23 (92.0)

Demographic and clinical characteristics as well as overall consent and satisfaction of all patients (*n *= 704) separated for diagnoses.

ICH, Intracranial hemorrhage; SAH, subarachnoid hemorrhage; GBS, Guillain-Barré syndrome; MG, myasthenia gravis; Neurodeg, neurodegenerative disease; Enceph, encephalopathy; Intox, intoxication; *n*, number; mRS, modified Rankin Scale.

**Figure 1:**
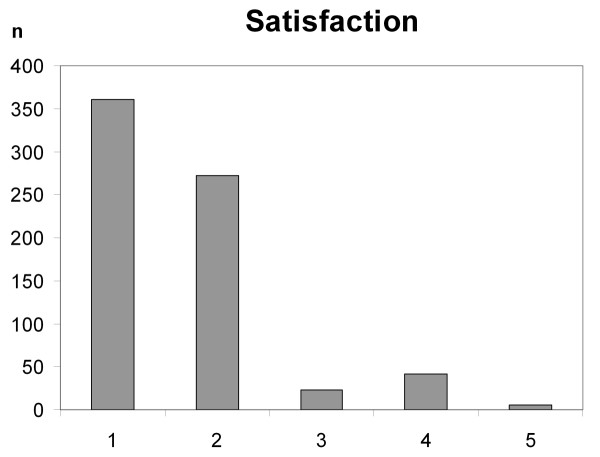
**Declared satisfaction, depending on the possible answers (very satisfied, satisfied, neither satisfied nor dissatisfied, dissatisfied, very dissatisfied)**.

Overall satisfaction was high (>90%) and did not depend on age, functional status before admission, sex, or the necessity of mechanical ventilation. Analysis of satisfaction according to diagnoses, however, revealed that patients with epilepsy were less satisfied than the general cohort (*P *< 0.01). The declared satisfaction depending on the possible answers is shown in Figure 1.

**Figure 2:**
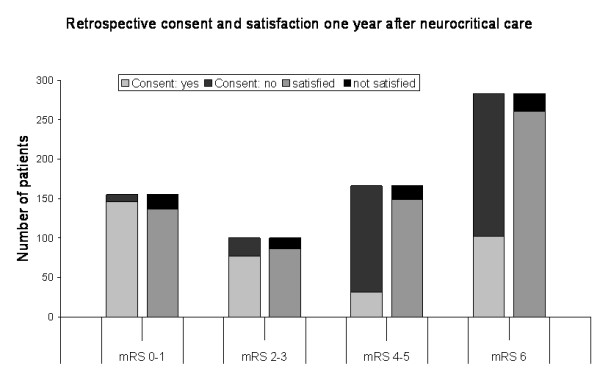
**Consent and satisfaction depending on functional outcome 1 year after discharge**. Although no differences in overall satisfaction were noted among the outcome groups, the χ^2 ^Test for retrospective consent revealed significant differences between the four groups (*P *< 0.001).

In contrast, retrospective consent was given in 51% of all patients only and was lowest in patients with stroke (*P *< 0.001; Table 2). The specific analysis of retrospective consent according to functional outcome is shown in Figure 2. Patients without disability (mRS 0 to 1), or who were only mildly to moderately disabled (mRS 2 to 3), consented to neurocritical care in 94%, and 76%, respectively, whereas patients with a mRS of 4 to 5 consented in only 19%. Relatives of patients who had died 1 year after disease onset gave retrospective consent in 38% (comparison between all groups: *P *< 0.001).

An outcome-based analysis of retrospective consent according to the specific diagnoses is given in Table 3. The logistic regression analysis of parameters that independently predicted consent is given in Table 4. Independent predictors for retrospective withdrawal of consent to neurocritical care were (a) worse functional outcome (expressed as an increase in the mRS), and (b) the diagnoses of ischemic and hemorrhagic stroke.

**Table 3:** Consent to treatment

	Ischemia	ICH	SAH	Meningoencephalitis	Epilepsy	GBS/MG	Neurodeg./ Enceph.	Cerebral neoplasm	Intox.
mRS 0-1 (*n*, %)	24/25 (96.0)	28/30 (93.3)	9/9 (100.0)	23/24 (95.87)	44/49 (89.8)	10/10 (100.0)	1/1 (100.0)	0/0	7/7 (100.0)
mRS 2-3 (*n*, %)	35/44 (79.9)	13/17 (76.5)	1/2 (50.0)	5/7 (71.4)	1/4 (25.0)	7/9 (77.8)	2/3 (66.7)	2/2 (100.0)	7/8 (87.5)
mRS 4-5 (*n*, %)	4/68 (5.8)	3/41 (7.3)	4/8 (50.0)	5/9 (55.6)	8/19 (42.1)	1/4 (25.0)	2/6 (33.3)	1/2 (50.0)	2/4 (50.0)
mRS 6 (*n*, %)	35/104 (33.7)	32/117 (27.4)	9/18 (50.0)	5/7 (71.4)	9/14 (64.3)	1/1 (100.0)	6/10 (60.0)	11/15 (73.3)	4/6 (66.7)

Consent to treatment (%) according to diagnosis and separated by functional outcome, that is, the proportion of patients with a specific disease and with a specific outcome who retrospectively agreed and consented to neurocritical care.

ICH, Intracranial hemorrhage; SAH, subarachnoid hemorrhage; GBS, Guillain-Barré syndrome; MG, myasthenia gravis; neurodeg, neurodegenerative disease; Enceph, encephalopathy; Intox, intoxication; *n*, number; mRS, modified Rankin Scale.

**Table 4:** Parameters predicting retrospective consent

	Consent to treatment OR (95% CI)	*P *value
**Univariate analysis**		

Demographic data		
Age	2.800 (0.350-5.808)	0.1057
Sex: female	3.407 (0.438-8.071)	0.1853
Mechanical ventilation	3.082 (0.414-22.923)	0.2716
Funct. status preadmission	1.263 (0.380-4.203)	0.7033
Diagnosis		
**Ischemia**	**0.440 (0.316-0.612)**	**<0.0001**
**ICH**	**0.441 (0.312-0.625)**	**<0.0001**
SAH	1.408 (0.689-2.880)	0.3482
**Meningoencephalitis**	**4.019 (1.908-8.470)**	**0.0003**
**Epilepsy**	**2.627 (1.581-4.363)**	**0.0002**
*GBS/MG*	*3.560 (1.313-9.650)*	*0.0126*
*Neurodeg./Encephalopathy*	*0.872 (0.764-1.031)*	*0.0219*
**Cerebral neoplasm**	**0.623 (0.453-0.824)**	**0.0038**
Intoxication	1.259 (0.853-1.738)	0.6432
Functional status at 1 year after discharge		
**mRS**	**0.588 (0.538-0.642)**	**<0.0001**

**Multivariate analysis**		

**Ischemia**	**0.294 (0.169-0.509)**	**<0.0001**
**ICH**	**0.306 (0.173-0.541)**	**<0.0001**
Meningoencephalitis	1.076 (0.426-2.721)	0.8764
Epilepsy	0.568 (0.274-1.179)	0.1289
GBS/MG	0.698 (0.218-2.230)	0.5426
Neurodeg./Encephalopathy	0.821 (0.691-1.368)	0.2574
**Cerebral neoplasm**	**0.572 (0.378-0.911)**	**0.0097**
**mRS**	**0.610 (0.555-0.671)**	**<0.0001**

Univariate and multivariate regression analysis to identify predisposing parameters for retrospective consent to neurocritical care. Parameters that reached significance (*P *< 0.01) are expressed in **bold**.

OR, odds ratio; CI, confidence interval; Funct, functional; ICH, intracranial hemorrhage; SAH; subarachnoid hemorrhage; GBS, Guillain-Barré syndrome; MG, myasthenia gravis; Neurodeg, neurodegenerative disease; mRS, modified Rankin Scale.

## Discussion

In this study, we investigated the frequency of retrospective agreement to the applied neurocritical care in correlation to neurologic disease and functional outcome. As a key finding, consent was high in patients with good functional outcome, whereas survival with a poor functional condition was related to the lowest rates of consent, especially in stroke patients.

First, it must noted that patients with acute onset of severe neurologic disease frequently are not capable of expressing their preferences regarding acceptance or decline of life-sustaining procedures [5,6]. Moreover, in the acute situation, a potentially existing advance directive may not be at hand [7], and relatives, if present, may be too distraught to participate appropriately in the decision process [8]. In addition, a dissociation of patients' and relatives' perception appears to exist in that regard, that survival itself does not necessarily imply survival with excellent outcome [9]. This may be caused partly by shortcomings during the informed-consent process for planned procedures [9].

Consent to neurointensive care measures the individual's satisfaction with being alive. Other studies of nonneurologic patients have shown that an age-related correlation might exist between older age and an incline in life satisfaction in women; however, this has not been revealed in men [10,11]. In our study, no correlation was found between consent and age or sex. Furthermore, several studies showed a relation between chronic neurologic illnesses and less life satisfaction [12-15]. As experienced, severe neurologic diseases, which led to neurointensive care, may have a long-term outcome similar to that of severe chronic diseases; this is in line with our results that more-severe long-term consequences lead to less retrospective consent.

The literature on the correlation of retrospective agreement to specific invasive treatment and functional outcome is less conclusive. Although Foerch and colleagues [16] reported a correlation between the retrospective decision against treatment and rather unfavorable outcome, and Walz and co-workers [17] also concluded that consent might depend on outcome in patients undergoing hemicraniectomy, other authors did not find a relation between functional outcome and retrospective agreement to therapy [18-20]. In another study, the willingness of patients to undergo intensive care to achieve even 1 additional month of survival was high; however, when asked if this would also apply if the patients were in a vegetative or severely neurologically impaired state, willingness to undergo intensive care was much less [21]. This again shows that there may be a difference in accepting general intensive care compared with neurointensive care, as the latter is associated with a higher likelihood of cognitive impairment.

Several novel issues presented here add to this discussion. Consent to neurocritical care declines with increasing disability. However, in patients who died in the course of the disease, increased rates of consent were presumed by the families. This reflects a previously unnoticed and highly important finding. These results indicate that death may not be judged to be the worst outcome by some patients or their relatives, and a proportion of patients may prefer death to severely disabled survival or a vegetative state [7,22-26].

The data presented here have several shortcomings. Data were collected in a single center, and the sample size may have limited the statistical power. Furthermore, because of intercultural differences, the results may not be internationally valid. In addition, consent was assessed one year after neurocritical care; however, answers given in questionnaires or telephone interviews have an inherent dependence on how the questions are stated, thus possibly leading to discrepancies across similar studies. Furthermore, other possibly important parameters, such as depression, were not collected. Finally, the fact that exclusively relatives have answered the questionnaires in all cases in which patients had died, represents a systemic bias, as the provision of time, money, and manpower when caring for patients at home *versus *transferring them into a nursing home may affect the given answers.

## Conclusions

Two aspects -- (a) incapability to state preferences regarding life-sustaining therapies after symptom onset and missing advance directive, and (b) absent consent to neurocritical care if survival is poor -- lead to a therapeutic dilemma in neurocritical care. Precisely because the majority of patients are not capable of stating their treatment preferences, a general initiation of neurocritical care assumes the patients' agreements in the acute phase [27-30]. This may result in prolonged disease duration and partly delays the decision to limit treatment until severe complications occur during the course of the disease [22,31,32]. The finding that patients consent to neurocritical care mainly if the functional outcome is favorable, or death, respectively, results in the following two issues that need further societal, ethical, and legal clarification: (a) efforts should be undertaken to achieve a greater public awareness of the necessity of advance directives, and (b) future investigations ultimately must focus on the identification of highly sensitive and specific predictors for outcome according to neurologic disease. Nonetheless, it will, in all likelihood, remain an ethical discussion whether to initiate neurocritical care in patients in whom palliative therapy may to be more appropriate.

## Key messages

• Retrospective consent in neurointensive care was in patients with good functional outcome, whereas survival with a poor functional condition was related to the lowest rates of consent, especially in stroke patients.

• Missing advance directives and assumed patients' agreements in the acute situation may lead to a general initiation of neurocritical care, which leads to prolonged disease duration.

## Abbreviations

GBS: Guillain-Barré syndrome; ICH: intracerebral hemorrhage; MG: myasthenia gravis; mRS: modified Rankin Scale; NICU: neurocritical care unit; SAH: subarachnoid hemorrhage.

## Competing interests

The authors declare that they have no competing interests.

## Authors' contributions

ICK and HBH designed the study and wrote the manuscript. ICK, JBK, LB, and CM obtained clinical data by reviewing institutional databases and the patient's medical charts. ICK and MK obtained outcome data by mailed questionnaires and telephone interviews. MK, PDS, and SS co-interpreted the data and critically reviewed the manuscript. All authors approved the final version of the manuscript.
